# Synchronous Gastric and Rectal Adenocarcinoma With Complete Response After Total Neoadjuvant Therapy: A Case Report

**DOI:** 10.7759/cureus.60787

**Published:** 2024-05-21

**Authors:** Daniela Martins, Rita Marques, Diana Martins, Ana Melo, João Pinto-de-Sousa

**Affiliations:** 1 General Surgery, Unidade Local de Saúde de Trás-Os-Montes e Alto Douro, Vila Real, PRT; 2 General Surgery, Clinical Academic Centre Trás-Os-Montes e Alto Douro, Vila Real, PRT; 3 Oncology, Unidade Local de Saúde de Trás-Os-Montes e Alto Douro, Vila Real, PRT; 4 Oncology, Clinical Academic Centre Trás-Os-Montes e Alto Douro, Vila Real, PRT

**Keywords:** neoadjuvant chemoradiotherapy, complete response, synchronous cancers, rectal cancer, gastric cancer

## Abstract

Synchronous gastric cancer with another neoplasm is a rare condition, with colorectal cancer being the most frequently associated neoplasm. This article presents a case of a 76-year-old male diagnosed with synchronous gastric and rectal cancer with complete remission of gastric and rectal neoplasms after adjuvant therapy. The patient exhibited symptoms prompting upper and lower endoscopies, revealing gastric and rectal adenocarcinomas, respectively. Staging was performed, and due to the locally advanced nature of both malignancies, the patient underwent total neoadjuvant therapy (TNT) for rectal cancer. The treatment consisted of external radiotherapy and neoadjuvant chemotherapy with oxaliplatin, leucovorin, folinic acid, and fluorouracil (FOLFOX). Remarkably, after seven cycles, a complete clinical response of the rectal neoplasm was achieved. Subsequent surgical resection included simultaneous subtotal gastrectomy and rectal anterior resection, resulting in a complete pathological response for both tumors. To the best of our knowledge, it is among the first cases to report a full pathological response in gastric cancer following TNT intended for rectal cancer.

## Introduction

Colorectal cancer ranks third, and gastric cancer ranks fifth in the global incidence of solid tumors, according to data from Global Cancer Statistics 2022 (GLOBOCAN 2022). As we delve into the complexities of malignancies, the occurrence of multiple primary malignancies (MPMs) adds another layer to the intricate landscape of cancer [1].

MPMs may be classified as synchronous or metachronous. Synchronous tumors are defined as MPMs that manifest within six months of the appearance of another tumor. On the other hand, a metachronous tumor will develop six months after another primary tumor [2-4].

Synchronous gastric cancer coexisting with another solid tumor is a rare condition, with an estimated incidence of 3.4%. The most frequently observed synchronous neoplasm is colorectal cancer, with a prevalence ranging from 20.1% to 37.2%, followed by lung cancer (12%) and liver cancer (11%) [2,5,6].

With the release of data from recent pivotal randomized controlled trials such as “Short-course radiotherapy followed by chemotherapy before total mesorectal excision (TME) versus preoperative chemoradiotherapy, TME, and optional adjuvant chemotherapy in locally advanced rectal cancer (RAPIDO): a randomised, open-label, phase 3 trial” (RAPIDO) and “Neoadjuvant chemotherapy with FOLFIRINOX and preoperative chemoradiotherapy for patients with locally advanced rectal cancer (UNICANCER-PRODIGE 23): a multicentre, randomised, open-label, phase 3 trial” (PRODIGE-23), total neoadjuvant therapy (TNT) has emerged as a leading approach in the treatment of locally advanced rectal cancer (LARC), and it is now recognized as a standard option for selected patients [7,8].

Treatment options for locally advanced gastric cancer, besides radical surgical resection, include neoadjuvant chemotherapy, radiation, various molecular-targeted therapies, and immunotherapy, specifically in cases of metastatic disease, all of which have a positive impact on survival [2].

Synchronous gastric and colorectal neoplasms pose a challenge for patient management. A multimodal approach including surgical and/or neoadjuvant/adjuvant therapy must be well-planned to enable a simultaneous procedure, ideally in a single surgical intervention, and to determine a chemotherapy scheme appropriate for both neoplasms.

To the best of our knowledge, it is among the first cases of synchronous gastric and rectal cancer with complete remission of both neoplasms, following TNT for LARC.

## Case presentation

Herein, the case of a 76-year-old male who underwent upper and lower endoscopies to investigate anemia (Hb 5.2g/dL) is presented. The patient was experiencing symptoms of asthenia, anorexia, and melena alternating with rectal bleeding, and had lost 6kg over four months.

He has a past medical history of hypertension, dyslipidemia, and atrial fibrillation, and he was under anticoagulation therapy with rivaroxaban. The patient didn’t have alcohol or smoking habits. On physical examination, he had a body mass index (BMI) of 29.8kg/m^2^, a normal painless abdominal palpation, and a rectal examination with melena and without palpable masses. He had no family history of cancer.

During the upper endoscopy, a 30mm suspicious ulcer was identified in the gastric antrum, and biopsy results indicated the presence of a gastric adenocarcinoma of the intestinal type. In the lower endoscopy, all the colon was visualized, and a circumferential ulcerated neoplasm in the rectum, located 12-15cm from the anal verge, was identified, with biopsy results confirming invasive adenocarcinoma.

**Figure 1:**
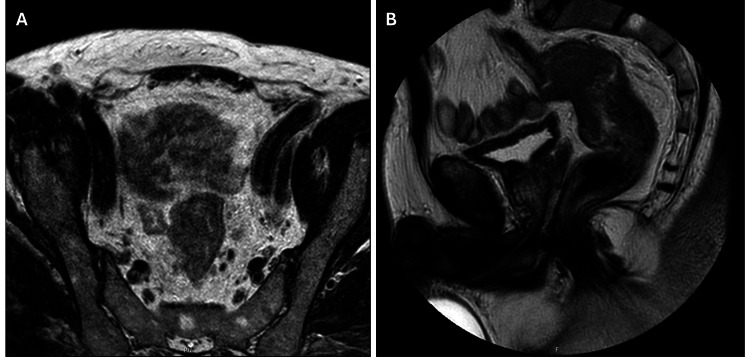
Pre-neoadjuvant therapy T2-weighted magnetic resonance images illustrating the extent and location of the rectal tumor. A - axial; B - sagittal

Pelvic magnetic resonance imaging (MRI) confirmed the presence of a rectal neoplasm, situated 10cm from the anal verge, exhibiting signs of transmural and extracapsular invasion spanning at least 10mm (Figure 1). The tumor exhibited contact but did not invade the anterior parietal reflection. Additionally, it showed separation from adjacent organs, with fewer than four lymph nodes showing suspected signs of metastasis. Staging thoraco-abdomino-pelvic computed tomography (CT) indicated the presence of lymph nodes near the celiac axis without any evidence of local or distant metastasis.

Based on these findings, the patient was diagnosed with a synchronous gastric adenocarcinoma staged as cT2N1M0 and a rectal adenocarcinoma staged as cT3cN2M0, following the tumor node metastasis classification (TNM) as per the 8th edition of American Joint Committee on Cancer Staging Manual [9]. Immunohistochemistry analysis of both tumors showed no microsatellite instability and there was no HER2 overexpression in gastric cancer tissue. Genetic germline testing was also performed and no hereditary pathogenic variants were identified.

**Figure 2:**
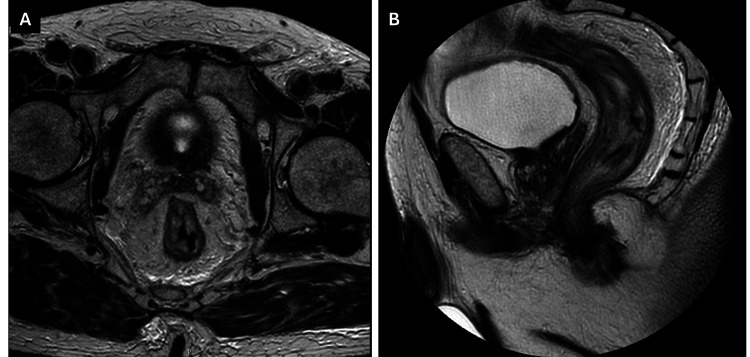
Post-neoadjuvant therapy T2-weighted magnetic resonance images demonstrating complete remission of the rectal tumor. A - axial; B - sagittal

The case was presented to the multidisciplinary board, and the patient was proposed for TNT, according to the RAPIDO protocol for rectal cancer, since it was the most advanced stage of neoplasm. The patient underwent external radiotherapy, with a total of 25Gy/5Fr over one week, followed by neoadjuvant chemotherapy with oxaliplatin, leucovorin, folinic acid, and fluorouracil (FOLFOX). After seven cycles of chemotherapy, MRI (Figure 2) and clinical evaluation revealed a complete clinical response of the rectal neoplasm, while thoracic and abdominal CT scans showed no evidence of progression in gastric cancer. The multidisciplinary tumor board proposed the patient to primary surgery and the patient was submitted to an open simultaneous subtotal gastrectomy with D2 lymphadenectomy and rectal anterior radical resection four weeks later. The procedure was uneventful, and the surgical team chose to not leave a defunctioning stoma.

Anatomopathological examination of the gastric specimen revealed a complete pathological response with negative margins and no evidence of disease in the 11 lymph nodes - ypT0N0 (0/11). Additionally, the pathologic report of the rectal specimen also revealed a complete tumor remission with clear margins and 19 negative lymph nodes - ypT0N0 (0/19).

Given the gastric nodal under-staging, the patient was submitted to a further five cycles of adjuvant chemotherapy with FOLFOX, to complete six months of peri-operative systemic treatment.

To date, after six months of follow-up, the patient remains without evidence of disease recurrence.

## Discussion

Managing synchronous neoplasms presents a challenge due to the lack of specific guidelines for approaching such scenarios [3,6].

While the mechanistic basis for the development of synchronous cancers remains unclear, potential contributing factors include lifestyle choices (use of alcohol, tobacco, and nitrosamines) and genetic factors. Defects in the mismatch repair system or the presence of microsatellite instability (MSI) may be linked to hereditary syndromes associated with a predisposition to multiple neoplasms, such as gastric and colorectal cancers. Additionally, the cell-cycle regulator p53, the DNA repair protein BRCA, and the CHEK2 gene have been implicated in the occurrence of multiple neoplasms. In this case report, no specific risk factors were identified, including genetic mutations or MSI, suggesting that there might be additional unidentified factors contributing to the development of multiple neoplasms [3,10].

Neoadjuvant chemotherapy with radiotherapy and subsequent TME, a commonly used multimodal treatment strategy for LARC, has improved its survival. TNT is one of these therapeutic approaches, supported by the National Comprehensive Cancer Network [11,12].

Individuals with LARC who undergo TNT may achieve a pathological complete response (pCR), which is defined as the absence of any remaining tumor cells in both the primary tumor site and the mesorectal lymph nodes. The rate of pCR after TNT is estimated at 40-60%, which positively affects the prognosis, improving survival [12,13].

For patients with LARC, the purpose of TNT is to boost the pre-surgery treatment by combining neoadjuvant chemotherapy and chemoradiotherapy (CRT). This aims to enhance the ability to surgically remove the tumor, increase the likelihood of achieving pCR, preserve organs, and ultimately improve survival rates. In the PRODIGE 23 randomized trial, the use of TNT including neoadjuvant chemotherapy with oxaliplatin, leucovorin, irinotecan, and 5-fluorouracil (FOLFIRINOX) and the conventionally administered CRT (followed by surgery and additional chemotherapy) showed improved rates of pCR, disease-free survival, and overall survival in comparison to the conventional neoadjuvant CRT (followed by surgery and additional chemotherapy) [8]. Another randomized trial, the RAPIDO trial, compared TNT (with short-course RT) with conventional CRT, both followed by neoadjuvant chemotherapy with CAPOX (capecitabine and oxaliplatin) or FOLFOX and revealed that the TNT regimen was associated with an improvement in pCR rate and a decrease in the occurrence of distant metastases at the five-year follow-up [7].

Regarding gastric cancer, survival rates have improved with the adoption of a combined strategy involving both surgery and perioperative chemotherapy. Neoadjuvant therapy increases the potential for tumor downstaging, enhances the likelihood of achieving negative margins, improves disease-free and overall survival, and facilitates the selection of patients who would benefit from a more aggressive surgical procedure. Additionally, it can lead to pCR, which typically falls within the range of 3-15% [14].

For the neoadjuvant treatment of gastric cancer (for stages ≥T2 or nodal metastatic involvement), chemotherapy with fluorouracil, leucovorin, oxaliplatin, and docetaxel (FLOT) (four cycles pre- and four cycles post-operative) is the standard of care for patients who are able to tolerate such a regimen. For patients who are unfit or who cannot tolerate the triplet regimen, a combination of fluoropyrimidine and platin (such as FOLFOX or CAPOX) is an option. FOLFOX or CAPOX are also options in TNT for rectal cancer, with potential effectiveness in treating gastric cancer as well.

Synchronous neoplasms establish a challenge for patient management requiring a multimodal approach of neoadjuvant or adjuvant treatment with surgical resection for MPMs, which, in this case, was applied with success. A good response to treatment from both neoplasms may mean that they have more molecular and genetic characteristics in common than previously thought.

In this case report, a pCR was achieved for both neoplasms, even though the neoadjuvant treatment was primarily directed at rectal cancer, thus posing many unanswered questions.

## Conclusions

This case report adds to the current knowledge about treating multiple heterogeneous synchronous neoplasms and, as far as we know, is one of the first clinical cases that reports a complete pathological response in a gastric cancer after TNT for rectal cancer.
